# Menstrual Cycle Length Changes Following Vaccination Against Influenza Alone or With COVID-19

**DOI:** 10.1001/jamanetworkopen.2025.7871

**Published:** 2025-04-29

**Authors:** Emily R. Boniface, Blair G. Darney, Agathe van Lamsweerde, Eleonora Benhar, Alexandra Alvergne, Alison Edelman

**Affiliations:** 1 Department of Obstetrics and Gynecology, Oregon Health & Science University, Portland; 2 OHSU-PSU (Oregon Health & Science University–Portland State University) School of Public Health, Portland; 3 Instituto Nacional de Salud Pública, Centro de Investigación en Salud Poblacional, Cuernavaca, México; 4 Natural Cycles Nordic AB, Stockholm, Sweden; 5 Institut des Sciences de l’Evolution de Montpellier, Montpellier University, Centre National de la Recherche Scientiﬁque, Institut de Recherche pour le Développement, Montpellier, France; 6 School of Anthropology and Museum Ethnography, University of Oxford, Oxford, UK

## Abstract

**Importance:**

Multiple studies have identified an association between COVID-19 vaccination and menstrual disturbances. Data on whether menstrual health is impacted by other vaccines are needed to counsel individuals about what to expect and to address vaccine hesitancy.

**Objective:**

To assess the association of changes in length of the menstrual cycle with influenza vaccination, with or without concurrent receipt of a COVID-19 vaccine.

**Design, Setting, and Participants:**

This global retrospective cohort study prospectively collected menstrual cycle data from April 25, 2023, to February 27, 2024 (4-5 cycles per individual), among international English-speaking users of a digital birth control application. Participants included individuals aged 18 to 45 years, not using hormonal contraception, and with average cycle lengths of 24 to 38 days in 3 consecutive cycles before receipt of vaccines.

**Exposure:**

Seasonal influenza vaccination with or without concurrent receipt of COVID-19 vaccine.

**Main Outcome and Measure:**

The primary outcome consisted of adjusted mean within-individual changes of menstrual cycle length assessed by vaccination group. Secondary analysis evaluated the phase of menstrual cycle at time of vaccination.

**Results:**

A total of 1501 individuals met the inclusion criteria, of whom 791 were vaccinated for influenza only and 710 were concurrently vaccinated for influenza and COVID-19. By race and ethnicity, 1 participant (0.1%) was American Indian or Alaska Native; 10 (0.7%), Asian; 3 (0.2%), Black; 15 (1.0%), Hispanic or Latina; 1 (0.1%), Middle Eastern or North African; 368 (24.5%), White; and 19 (1.3%), other; and 1084 (72.2%), missing. Most of the cohort was younger than 35 years (1230 [82.0%]), had at least a college degree (1122 [74.8%]), and was located in the US or Canada (938 [62.5%]). Individuals vaccinated for influenza alone experienced an adjusted mean increase of 0.40 (95% CI, 0.08-0.72) days, while those vaccinated concurrently for influenza and COVID-19 experienced a mean increase of 0.49 (95% CI, 0.16-0.83) days (*P* = .69 for difference between vaccine groups). A total of 37 individuals (4.7%) experienced a change in cycle length of at least 8 days with influenza vaccine only and 42 (5.9%) with concurrent receipt of both vaccines (*P* = .28). In the postvaccination cycle, both vaccination groups returned to their prevaccination cycle lengths. Menstrual cycle changes occurred with vaccination in the follicular phase but not the luteal phase.

**Conclusions and Relevance:**

In this cohort study of individuals with regular menstrual cycles, influenza vaccine given alone or in combination with a COVID-19 vaccine was associated with a small but temporary change in menstrual cycle length. These findings may help clinicians confirm the utility of vaccination for patients with concerns about menstrual adverse effects of vaccination.

## Key Points

**Question:**

Is the influenza vaccine alone or with a COVID-19 vaccine associated with change in menstrual cycle length?

**Findings:**

In this cohort study of 1501 participants, in individuals with regular menstrual cycles, a temporary small but statistically significant increase in menstrual cycle length (<1 day) with receipt of an influenza vaccine, with or without a COVID-19 vaccine, based on vaccination in the follicular phase was found.

**Meaning:**

These findings may help to confirm the utility of vaccination for individuals with concerns about adverse effects of vaccination on menstruation.

## Teaser

This cohort study compares changes in menstrual cycle length among individuals with regular prevaccination menstrual cycles who received influenza vaccination alone or concurrently with COVID-19 vaccination.

## Introduction

In January 2019, the World Health Organization recognized vaccine hesitancy as one of the top 10 threats to global health.^1^ The public’s concerns about vaccines and their adverse effects can directly impact vaccine hesitancy and impede the uptake of vaccines, which in turn increases rates of preventable disease.^2,3,4^ Reported menstrual changes following receipt of the COVID-19 vaccine received a substantial amount of attention and concern from civil society and the media, which impacted uptake.^5^ However, establishment of a link between vaccines and menstrual changes is not new. For example, the Japanese government was forced to suspend their human papillomavirus (HPV) vaccine program in 2013 following reports of adverse effects including menstrual cycle disturbances in adolescents and young women, which raised fears of potential impacts on future fertility.^6^ Initial uptake was 70%, but following these reports, vaccination uptake plummeted to less than 1% among the eligible population.^7,8,9^ The absence of data to directly address anecdotal reports has had long-lasting impacts: uptake of HPV vaccine is still low in Japan, and HPV vaccine programs in other countries were also shown to be negatively impacted.^10,11,12^ More importantly, the lack of HPV vaccine uptake is estimated to result in approximately 10 000 preventable deaths from cervical cancer in Japan in the next 50 years.^13^

Prior work from some of the investigators from the present study and others^14,15,16,17^ has found a small temporary change in menstrual cycle length and heaviness in menstrual flow for individuals receiving the COVID-19 vaccine, particularly for those who are vaccinated during the follicular phase of the menstrual cycle. While the underlying mechanisms behind this finding are not fully understood, we have known for several decades that the immune and reproductive systems interact closely with one another, but the interaction does not cause infertility.^18^ An individual’s response to a vaccine is impacted by a variety of factors, including prior exposure to the disease and/or vaccine, immunogenicity of the vaccine, time from either event, and biological sex, among others.^19,20^ Despite the potential for menstrual cycle disturbances following vaccination, menstrual health outcomes have been overlooked in prior vaccine clinical trials, creating a critical knowledge gap about these important preventative health tools.^16^

Influenza is a long-standing common endemic virus. Influenza vaccination is the best way to prevent or decrease complications of influenza and is recommended annually to offset waning immunity. Now that COVID-19 infection has transitioned from pandemic to endemic, we appear to be moving into an annual vaccination schedule that will include recommendations to receive both influenza and COVID-19 vaccines. However, any potential impact of influenza vaccination on menstrual cycle changes and whether those changes may differ with concurrent receipt of the COVID-19 vaccine remain unknown. Herein we analyze prospectively collected menstrual cycle data among those who received the influenza vaccine alone or on the same day as COVID-19 vaccination. We compare changes in menstrual cycle length in days and the prevalence of clinically meaningful changes (≥8 days) between vaccination groups,^21^ in both the vaccination and postvaccination cycles.

## Methods

### Data Source

**Figure 1.**
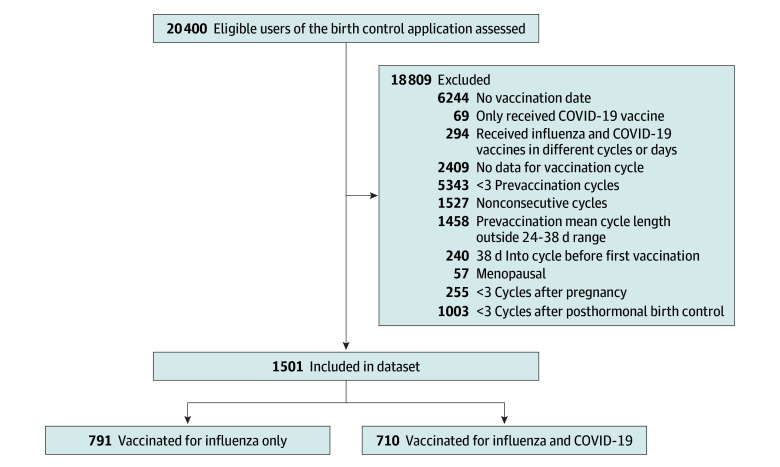
Sample Flow Diagram

This retrospective cohort study used prospectively collected menstrual cycle data from a digital birth control application (Natural Cycles; Nordic AB). Individuals use the application to plan or prevent pregnancy without the use of hormonal contraceptive methods; details about the variables collected by the application have been published previously.^22^ To be eligible for study inclusion, individuals needed to consent to the use of their deidentified data for research purposes, be aged 18 to 45 years, and respond to an in-application message about receipt and timing of a seasonal influenza and/or COVID-19 vaccination in August 2023 or later. Retrospective self-reported data on vaccination timing were then paired with prospectively collected data on menstrual cycles. We excluded individuals who indicated that they had received at least 1 vaccination but did not provide a vaccination date, only received COVID-19 vaccine, received both vaccines but in different cycles or different days within the same cycle, had no data for the vaccination cycle or fewer than 3 prevaccination cycles, had nonconsecutive cycles, had a mean prevaccination cycle length outside the reference range of 24 to 38 days,^21^ were at least 38 days into the vaccination cycle prior to receiving a vaccine, self-identified as menopausal, or were less than 3 cycles after pregnancy or after hormonal contraception use for the entire study period (Figure 1). The Institutional Review Board of the Oregon Health & Science University, Portland, approved the protocol and did not require informed written consent beyond an introduction to the brief survey and a reminder regarding the revokable consent to the use of deidentified data for research purposes provided by users within the application. This report follows the Strengthening the Reporting of Observational Studies in Epidemiology (STROBE) reporting guideline for cohort studies.

Cycle data in the final analytical sample ranged between April 25, 2023, and February 27, 2024; vaccines were received from August 21, 2023, to January 29, 2024. Each individual contributed data from a minimum of 4 consecutive cycles: 3 prevaccination cycles and the cycle in which they received their vaccines (vaccination cycle). We also included data from a fifth cycle immediately following the vaccination cycle (postvaccination cycle) if available. If data from the postvaccination cycle were not available, we excluded those individuals (n = 30) from postvaccination cycle analyses.

Our primary binary independent variable was vaccination group: receipt of an influenza vaccine only or concurrent receipt of both influenza and COVID-19 vaccines on the same day. We chose to focus on these study groups since completely naive (unvaccinated) individuals are rare in the study population. Our primary outcome was the adjusted within-individual change in menstrual cycle length (in days) from the mean of the 3 prevaccination cycles to the vaccination cycle; each individual therefore served as their own control. We also assessed the change in cycle length from the prevaccination cycle mean to the postvaccination cycle, and whether the vaccination or postvaccination change in cycle length was clinically meaningful (defined as a change of ≥8 days).^21^

We included several sociodemographic characteristics collected within the birth control application. We categorized age into approximate 5-year groups: 18 to 24, 25 to 29, 30 to 34, 35 to 39, and 40 to 45 years. Individuals reported their race and ethnicity using options defined by the application as American Indian or Alaska Native, Asian, Black, Hispanic or Latina, Middle Eastern or North African, White, or other group. We reported racial and ethnic categories to characterize this sample population but did not include these data in our primary adjusted model. We used categorical variables for body mass index (BMI; calculated as the weight in kilograms divided by the height in meters squared): underweight (<18.5), normal weight (18.5-24.9), overweight (25.0-29.9), and obesity (≥30.0). We classified geographic location as the UK and Channel Islands, continental Europe, US and Canada, and other regions. We also used binary variables to characterize parity (nulliparous vs parous), educational level (less than a college degree vs college degree or more), and relationship status (in a relationship vs not in a relationship). Notably, the birth control application’s sociodemographic data collection patterns have changed over time, and report of many sociodemographic variables is optional within the application, which resulted in a large degree of missingness for several variables.

### Statistical Analysis

We compared all sociodemographic characteristics by vaccination group using a Pearson χ^2^ or Fisher exact test. We calculated the change in cycle length from the prevaccination mean to the vaccination and postvaccination cycles (excluding 30 individuals with no data for the postvaccination cycle) and adjusted the estimates using linear regression models with change in cycle length as the outcome, vaccination group as the primary independent variable, and age group, BMI category, and parity as adjusting covariates. We then graphed the mean marginal change in cycle length from the models with 95% CIs for individuals who only received the influenza vaccine and those who received both the influenza and COVID-19 vaccine on the same day. We used multiple imputation by chained equations^23^ with 50 rounds of imputation to address missingness in adjusting covariates. Data missingness was a function of changes to demographic data collection by the application and considered missing at random. We compared the percentage of individuals who experienced a clinically meaningful change in cycle length (≥8 days) during the vaccination cycle across vaccination groups using a Pearson χ^2^ test. We then repeated this analysis for the postvaccination cycle among those who had experienced a clinically meaningful change in cycle length during the prior vaccination cycle.

In addition to assessing unadjusted changes in cycle length, we conducted several sensitivity analyses to confirm the robustness of our results for both the vaccination and postvaccination cycles. First, we excluded any individuals who reported polycystic ovary syndrome, thyroid disorder, or endometriosis (n = 145). Second, we excluded anyone who reported use of emergency contraception in any cycle during the study period (n = 58). Third, we excluded anyone with at least 1 prevaccination cycle outside the 24- to 38-day range (n = 247). Fourth, we developed multivariable models with additional adjusting covariates: race and ethnicity (collapsed to White compared with some other group due to sample size), global region (collapsed to US and Canada compared with other regions), educational level, and relationship status. Finally, we assessed the adjusted change in cycle length based on the menstrual phase timing of vaccination for both vaccination groups: receipt of vaccines in the follicular phase (first day of the cycle through the day of ovulation as estimated by the application’s validated algorithm) or luteal phase (day after ovulation through the last day of the cycle), using methods described previously.^24^ All analyses were conducted using Stata, version 17.0 (StataCorp LLC), and 2-sided *P* ≤ .05 indicated statistical significance.

## Results

Among the 1501 individuals in this analytical cohort, 1230 (82.0%) were younger than 35 years; 1122 (74.8%) had at least a college degree; and 938 (62.5%) were located in the US or Canada (Table 1). By race and ethnicity, 1 participant (0.1%) was American Indian or Alaska Native; 10 (0.7%), Asian; 3 (0.2%), Black; 15 (1.0%), Hispanic or Latina; 1 (0.1%), Middle Eastern or North African; 368 (24.5%), White; and 19 (1.3%), other; and 1084 (72.2%), missing. Figure 1 summarizes participant flow. The study sample included 791 individuals vaccinated for influenza only and 710 vaccinated for both influenza and COVID-19 on the same day, representing a total of 7475 cycles, with 30 individuals missing data from the postvaccination cycle. Compared with those who received only the influenza vaccine, individuals who received both vaccines were more likely to be older than 30 years (391 [55.1%] vs 346 [43.7%]; *P* < .001) and more likely to be located in the US or Canada (511 [72.0%] vs 427 [54.0%]; *P* < .001). All missing data are tabulated in Table 1.

**Table 1.** Characteristics of Study Participants

Characteristic	Vaccine group, No. (%)			*P* value
	Influenza only (n = 791)	Influenza and COVID-19 (n = 710)	All (N = 1501)	
Age, y				
18-24	147 (18.6)	70 (9.9)	217 (14.5)	<.001
25-29	298 (37.7)	249 (35.1)	547 (36.4)	
30-34	211 (26.7)	255 (35.9)	466 (31.0)	
35-39	108 (13.7)	111 (15.6)	219 (14.6)	
40-45	27 (3.4)	25 (3.5)	52 (3.5)	
Race and ethnicity				
American Indian or Alaska Native	1 (0.1)	0	1 (0.1)	.01
Asian	5 (0.6)	5 (0.7)	10 (0.7)	
Black	2 (0.3)	1 (0.1)	3 (0.2)	
Hispanic or Latina	7 (0.9)	8 (1.1)	15 (1.0)	
Middle Eastern or North African	1 (0.1)	0	1 (0.1)	
White	174 (22.0)	194 (27.3)	368 (24.5)	
Some other race^a^	11 (1.4)	8 (1.1)	19 (1.3)	
Missing	590 (74.6)	494 (69.6)	1084 (72.2)	
Parity				
Nulliparous	214 (27.1)	245 (34.5)	459 (30.6)	.003
Parous	34 (4.3)	19 (2.7)	53 (3.5)	
Missing	543 (68.6)	446 (62.8)	989 (65.9)	
BMI				
Underweight (<18.5)	17 (2.1)	10 (1.4)	27 (1.8)	.12
Normal weight (18.5-24.9)	325 (41.1)	282 (39.7)	607 (40.4)	
Overweight (25.0-29.9)	165 (20.9)	122 (17.2)	287 (19.1)	
Obesity (≥30.0)	81 (10.2)	80 (11.3)	161 (10.7)	
Missing	203 (25.7)	216 (30.4)	419 (27.9)	
Educational level				
Less than college degree	61 (7.7)	31 (4.4)	92 (6.1)	.02
College degree or more	589 (74.5)	533 (75.1)	1122 (74.8)	
Missing	141 (17.8)	146 (20.6)	287 (19.1)	
Relationship status				
Not in relationship	32 (4.0)	34 (4.8)	66 (4.4)	.03
In relationship	214 (27.1)	233 (32.8)	447 (29.8)	
Missing	545 (68.9)	443 (62.4)	988 (65.8)	
Geographic region				
UK and Channel Islands	266 (33.6)	168 (23.7)	434 (28.9)	<.001
Europe	96 (12.1)	30 (4.2)	126 (8.4)	
US and Canada	427 (54.0)	511 (72.0)	938 (62.5)	
Other	2 (0.3)	1 (0.1)	3 (0.2)	

Abbreviation: BMI, body mass index (calculated as weight in kilograms divided by height in meters squared).

^a^ Individuals in this category selected “other” when reporting their race and ethnicity within the application; no additional data are available regarding their racial and ethnic identities.

**Figure 2.**
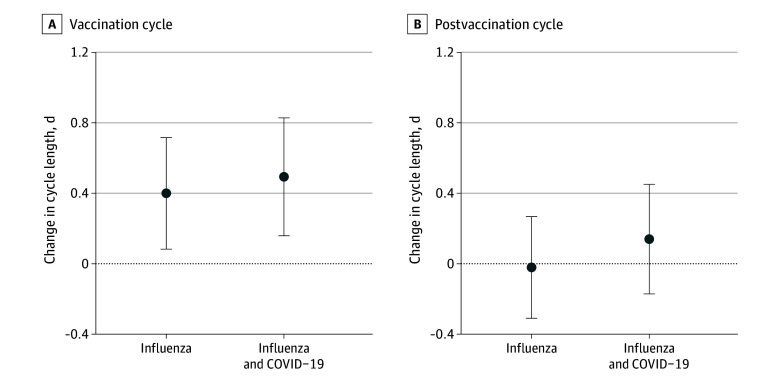
Adjusted Change in Menstrual Cycle Length by Vaccination Group Change in cycle length was calculated as the difference, in days, from the prevaccination mean cycle length to the vaccination cycle (N = 1501) and the postvaccination cycle (n = 1471). Individuals were vaccinated for influenza alone or influenza concurrently with COVID-19. Estimates were adjusted for age group, body mass index category, and parity following multiple imputation for missing data. Error bars represent 95% CIs; the dotted horizontal line at 0 indicates no change from the prevaccination period.

Both vaccination groups experienced a small but statistically significant adjusted increase in cycle length during the vaccination cycle (Figure 2A). Individuals vaccinated for influenza alone experienced a mean increase of 0.40 (95% CI, 0.08-0.72) days, while those vaccinated concurrently for influenza and COVID-19 experienced a mean increase of 0.49 (95% CI, 0.16-0.83) days (*P* = .69 for difference between vaccine groups). In the postvaccination cycle, neither group experienced cycle lengths that were significantly different from those in their prevaccination period (Figure 2B). The adjusted mean change in cycle length was −0.02 (95% CI, −0.31 to 0.27) days for the influenza only group and 0.14 (95% CI, −0.17 to 0.45) days for the influenza and COVID-19 group (*P* = .46 for the between-group difference). eTable 1 in Supplement 1 includes summary statistics for cycle lengths and changes from prevaccination mean.

During the vaccination cycle, the percentage of individuals who experienced a clinically meaningful change in cycle length of 8 days or more was slightly higher in the group that received both vaccines concurrently compared with influenza only (42 of 710 [5.9%] vs 37 of 791 [4.7%]) (Table 2), but the difference was not statistically significant (*P* = .28). Among those who experienced a clinically meaningful change in cycle length during the vaccination cycle (≥8 days), there were no statistically significant differences in the percentage who continued to experience a clinically meaningful change in the postvaccination cycle between the vaccination groups (10 of 36 [27.8%] for influenza only vs 8 of 39 [20.5%] for both vaccines; *P* = .46).

**Table 2.** Percentage of Individuals Experiencing a Change in Cycle Length of 8 Days of More From the Mean Prevaccination to Vaccination Cycles or Postvaccination Cycle

Cycle	Outcome	Vaccination group, No./total No. (%)		*P* value
		Influenza only	Influenza and COVID-19	
Vaccination	≥8 d Change	37/791 (4.7)	42//710 (5.9)	.28
Postvaccination	≥8 d Change^a^	10/36 (27.8)	8/39 (20.5)	.46

^a^ Among individuals with the clinically meaningful change in cycle length (≥8 days) in the vaccination cycle. Individuals with no data available for the postvaccination cycle (n = 4) were excluded from postvaccination cycle tabulations.

Our sensitivity analyses excluding individuals with polycystic ovary syndrome, thyroid disorder, or endometriosis, individuals reporting emergency contraception use, or individuals with any prevaccination cycle lengths outside the normal range or adjusting for the full set of covariates did not alter our findings in a meaningful way, with the exception that the change in vaccination cycle length for individuals vaccinated for influenza alone was not statistically significant after excluding those with self-reported gynecological or thyroid disorders (eTable 2 in Supplement 1). However, in that sensitivity analysis, the cycle length change was significant for individuals who received both vaccines with an increase of 0.53 (95% CI, 0.21 to 0.86) days.

**Figure 3.**
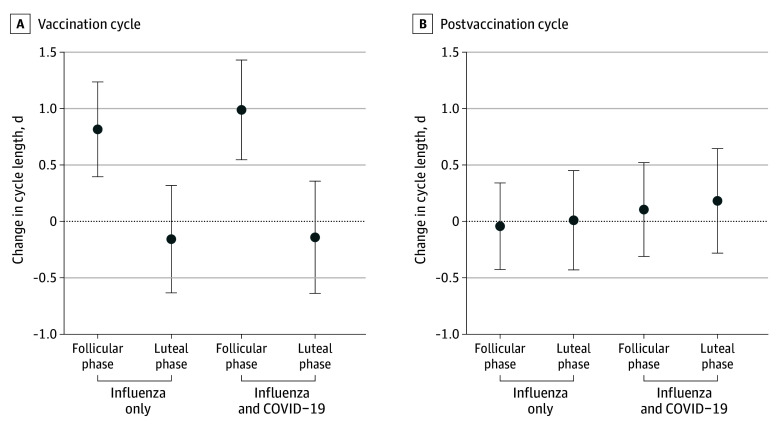
Adjusted Change in Menstrual Cycle Length, by Vaccination Group and Menstrual Phase of Vaccination Change in cycle length was calculated as the difference in days from the prevaccination mean cycle length to the vaccination cycle and the postvaccination cycle, by vaccination group and timing of vaccination (follicular vs luteal phase). Individuals were vaccinated for influenza alone (457 in the follicular phase and 334 in the luteal phase) or influenza and COVID-19 concurrently (395 in the follicular phase and 315 in the luteal phase). Estimates were adjusted for age group, body mass index category, and parity following multiple imputation for missing data. Error bars represent 95% CIs; the dotted horizontal line at 0 indicates no change from the prevaccination period.

When we examined the adjusted change in cycle length in both vaccination groups by the menstrual phase of vaccination, only individuals who were vaccinated in the follicular phase experienced a statistically significant increase in cycle length compared with their prevaccination mean length: increase of 0.82 (95% CI, 0.40-1.24) days for influenza alone and 0.99 (95% CI, 0.55-1.43) days for concurrent influenza and COVID-19 vaccines (Figure 3A). Individuals vaccinated in the luteal phase experienced no change in cycle length: −0.16 (95% CI, −0.63 to 0.32) days for influenza alone vs −0.14 (95% CI, −0.64 to 0.36) days for both vaccines. In the postvaccination cycle, no groups experienced a significant change in cycle length regardless of menstrual phase of vaccination or vaccines received (Figure 3B).

## Discussion

Misinformation and the dearth of data to confirm or refute the vaccine experience can decrease acceptability and uptake of a vaccine. Prior work including investigators from the present study^14,15^ found that the COVID-19 vaccine temporarily lengthens the menstrual cycle by about 1 day or less, while a small subset of individuals will experience a clinically meaningful cycle length change of 8 days or more. In the present cohort study, we found a similarly temporary small increase in cycle length for individuals receiving seasonal influenza vaccine only or influenza plus COVID-19 vaccine, based on vaccination during the follicular phase, and a small subset who experienced a cycle length change of 8 days or more. This provides an important first data point about how influenza vaccination might affect menstrual cyclicity, a topic that has been largely ignored throughout the almost century-long history of influenza vaccines.

A substantial amount of one’s lifetime is spent menstruating. It is a common routine bodily function occurring for approximately 1 week each month for 40 years. While the COVID-19 pandemic brought many challenges, it did highlight the lack of evidence on this important patient-oriented outcome. Public concern about new vaccines creates mistrust about all vaccines. We have seen a recent decline in overall vaccination uptake.^25^ We hypothesized that given the endemic nature of influenza and the widespread exposure to the influenza vaccine, we might not see any signal, but given that vaccines are meant to cause an immune response each time they are received, it is also not surprising that our findings are similar to those for the COVID-19 vaccine. Notably, our findings also suggest that concurrent administration of the COVID-19 vaccine with influenza vaccination does not appear to significantly increase the risk of menstrual cycle disturbances, which may help clinicians confirm the utility of vaccination with these temporary changes and help improve vaccine uptake rates for both endemic diseases.

The increase in cycle length we observed appears to be based on individuals vaccinated in the follicular phase of their menstrual cycle. This is in line with previous work from some of the investigators of the present study,^24^ which found an approximately 1-day increase in cycle length for individuals vaccinated for COVID-19 during the follicular phase, but no change for those vaccinated in the luteal phase or for an unvaccinated control group. Our results support the current hypothesis that the immune response triggered by vaccination temporarily impacts the hypothalamic-pituitary-ovarian axis, although it is unclear whether this is a series of temporary responses or 1 primary change and at what level of the axis this occurs.^24,26,27^ Individuals who are concerned about potential menstrual cycle disturbances following vaccination for influenza and/or COVID-19 could consider timing their vaccination to coincide with their luteal phase to minimize their risk of cycle length changes.

While small changes in menstrual health may not seem meaningful to many clinicians and scientists, any perceived impact in a routine bodily function linked to fertility can cause alarm and contribute to vaccine hesitancy. To draw a parallel, this might be comparable to whether reports of temporary erectile dysfunction occurred post vaccination, which is by no means a serious adverse event but is a cause of distress if unanticipated, potentially raising concerns for future fertility, and which could certainly fuel vaccine hesitancy. While sporadic deviations from menstrual norms are not cause for clinical concern, they can have a large adverse impact on the quality of life during menstruation for individuals who experience episodes of social embarrassment, anxiety related to uncontained bleeding or pregnancy, and worry about what bleeding changes mean for their overall health and fertility.^28,29,30^ Any change, even if small and not clinically relevant, is important to the public, and even more so in the context of vaccines and rampant misinformation.^31^

### Strengths and Limitations

The strengths of our study include a large global sample of prospectively collected menstrual cycle data before, during, and after self-confirmed vaccine timing and type. Menstrual cycles are known to be inherently variable, but we attempted to mitigate this by using data from individuals not using hormonal contraception with proven regular cycles prior to vaccination as their own controls and excluding individuals with known irregular cycles.

This study also has some limitations. First, our dataset had high levels of missingness for several sociodemographic characteristics, potentially limiting our ability to address confounding. However, various approaches to multiple imputation did not change our findings. Second, our sample is largely White, nulliparous, and highly educated and has a low BMI, which could limit the generalizability of our results. Third, we were only able to adjust for sociodemographic characteristics collected by the application; our estimates may be affected by residual confounding. Fourth, vaccination dates were self-reported and may be subject to recall bias, but we conducted the survey during the most active time of influenza vaccination. We also excluded a large proportion of eligible individuals who did not have enough cycle data to adequately assess our outcome. This is due to the fact that the user base for the birth control application has grown over time, and newer users were not yet tracking their cycle data around the time of vaccination. Prior work^14,15,17,24^ compared COVID-19 vaccinated individuals with an unvaccinated control group; in this study we compare receipt of influenza vaccine alone or in combination with COVID-19 vaccine, which represents a common clinical scenario. Individuals truly naive to either vaccine are rare; both influenza and the influenza vaccine have been endemic for decades.

## Conclusions

In this cohort study, we found that receipt of influenza vaccine alone and receipt of both influenza and COVID-19 on the same day were associated with small (<1 day) changes in menstrual cycle length, based on vaccination during the follicular phase. We further showed no differences by vaccination group in the proportion of individuals who experience a clinically meaningful (≥8 days) change in cycle length. Our findings can confirm that concurrent receipt of influenza and COVID-19 vaccines does not appear to be associated with large menstrual cycle changes in most people.

## Notes

Supplement 1. **eTable 1.** Summary Statistics for Change in Cycle Length for Vaccination and Postvaccination Cycles, Overall and by Vaccination Group **eTable 2.** Sensitivity Analyses for the Primary Outcome of the Change in Cycle Length, in Days, From the Prevaccination Mean to the Vaccination and Postvaccination Cycles, by Vaccination Status

Supplement 2.
Data Sharing Statement
