# Supplementary material for: Menstrual Cycle Length Changes Following Vaccination Against Influenza Alone or With COVID-19
Source: JAMA Netw Open. 2025 Apr 29;8(4):e257871. doi: 10.1001/jamanetworkopen.2025.7871 (PMC12042056; doi:10.1001/jamanetworkopen.2025.7871)
Supplement: Supplement 1. — eTable 1. Summary Statistics for Change in Cycle Length for Vaccination and Postvaccination Cycles, Overall and by Vaccination Group eTable 2. Sensitivity Analyses for the Primary Outcome of the Change in Cycle Length, in Days, From the Prevaccination Mean to the Vaccination and Postvaccination Cycles, by Vaccination Status [file jamanetwopen-e257871-s001.pdf]

Supplementary Online Content

Boniface ER, Darney BG, van Lamsweerde A, Benhar E, Alvergne A, Edelman A. Menstrual cycle length changes following vaccination against influenza alone or with COVID-19. *JAMA Netw Open.* 2025;8(4):e257871. doi:10.1001/jamanetworkopen.2025.7871

eTable 1. Summary Statistics for Change in Cycle Length for Vaccination and Postvaccination Cycles, Overall and by Vaccination Group

eTable 2. Sensitivity Analyses for the Primary Outcome of the Change in Cycle Length, in Days, From the Prevaccination Mean to the Vaccination and Postvaccination Cycles, by Vaccination Status

This supplementary material has been provided by the authors to give readers additional information about their work.

© 2025 Boniface ER et al. *JAMA Network Open*.

© 2025 Boniface ER et al. *JAMA Network Open*.

eTable 1. Summary Statistics for Change in Cycle Length for Vaccination and Postvaccination Cycles, Overall and by Vaccination Group

Outcome (days)	Statistic	Influenza	Influenza & COVID-19	Overall
Vaccination cycle length	Mean ± SD	29.2 ± 4.6	29.2 ± 5.3	29.2 ± 4.9
	Range	18 – 79	16 – 97	16 – 97
Change from pre-vaccination period to vaccination cycle	Mean ± SD	0.39 ± 3.96	0.51 ± 4.95	0.44 ± 4.45
	Range	-15 – 46	-20 – 70.7	-20 – 70.7
Post-vaccination cycle length	Mean ± SD	28.8 ± 4.6	28.8 ± 4.7	28.8 ± 4.7
	Range	16 – 69	15 – 70	15 – 70
Change from pre-vaccination period to post-vaccination cycle	Mean ± SD	-0.01 ± 4.03	0.12 ± 4.14	0.05 ± 4.08
	Range	-21 – 33.3	-17 – 36.3	-17 – 36.3

eTable 2. Sensitivity Analyses for the Primary Outcome of the Change in Cycle Length, in Days, From the Prevaccination Mean to the Vaccination and Postvaccination Cycles, by Vaccination Status

Analysis	Vaccination Cycle		Post-vaccination cycle		n excluded
	Flu	Flu & COVID	Flu	Flu & COVID	
Unadjusted	0.39 (0.08 – 0.70)	0.51 (0.18 – 0.83)	-0.01 (-0.29 – 0.28)	0.12(-0.18 – 0.43)	0
Excluding anyone with PCOS*, thyroid disorder, or endometriosis	0.25 (-0.07 – 0.56)	0.53 (0.21 – 0.86)	0.08 (-0.21 – 0.38)	0.10 (-0.22 – 0.41)	140
Excluding anyone who used emergency contraception during study cycles	0.42 (0.10 – 0.73)	0.49 (0.16 – 0.82)	-0.03 (-0.32 – 0.26)	0.15 (-0.16 – 0.47)	56
Excluding anyone with any pre-vaccination cycle outside the 24-38 day range	0.47 (0.17 – 0.77)	0.57 (0.26 – 0.89)	0.14 (-0.13 – 0.41)	0.26 (-0.03 – 0.55)	231
Fully adjusted**	0.41 (0.09 – 0.74)	0.48 (0.14 – 0.82)	-0.05 (-0.35 – 0.24)	0.18 (-0.14 – 0.49)	0

* PCOS = Polycystic ovary syndrome
** Adjusted for age group, BMI category, parity, race and ethnicity, geographic region, education level, and relationship status following multiple imputation with chained equations.

© 2025 Boniface ER et al. *JAMA Network Open*.
