# Supplementary material for: Menstrual Cycle Length Changes Following Vaccination Against Influenza Alone or With COVID-19
Source: JAMA Netw Open. 2025 Apr 29;8(4):e257871. doi: 10.1001/jamanetworkopen.2025.7871 (PMC12042056; doi:10.1001/jamanetworkopen.2025.7871)
Supplement: Supplement 2. — Data Sharing Statement [file jamanetwopen-e257871-s002.pdf]

Data Sharing Statement

Boniface. Menstrual Cycle Length Changes Following Vaccination Against Influenza Alone or With COVID-19. *JAMA Netw Open*. Published April 29, 2025.
doi:10.1001/jamanetworkopen.2025.7871

Data
Data available: No

Additional Information
Explanation for why data not available: The data used for this study are available from Natural Cycles and were used under a data use agreement. Restrictions apply to their availability. We do not own the dataset. We have a datasharing agreement with the menstrual tracking digital platform to access the dataset. We can supply the data dictionary on request after receiving a datasharing agreement and review of regulatory documents
